# LncRNA UCA1 mediates Cetuximab resistance in Colorectal Cancer via the MiR-495 and HGF/c-MET Pathways

**DOI:** 10.7150/jca.65687

**Published:** 2022-01-01

**Authors:** Heng-heng Yuan, Xin-chen Zhang, Xiao-li Wei, Wen-jie Zhang, Xiao-xue Du, Peng Huang, Hao Chen, Lu Bai, Hong-feng Zhang, Yu Han

**Affiliations:** 1 Department of Gastrointestinal Oncology, Harbin Medical University Cancer Hospital, Harbin, Heilongjiang Province, China.; 2 Department of General Surgery, The Second Affiliated Hospital of Harbin Medical University, Harbin, Heilongjiang Province, China.; 3 Department of Gastric Surgery, Harbin Medical University Cancer Hospital, Harbin, Heilongjiang Province, China.

**Keywords:** UCA1, cetuximab, metastatic colorectal cancer, miR-495, hepatocyte growth factor/c-MET signalling pathway

## Abstract

**Background:** Cetuximab is one of the most widely used monoclonal antibodies to treat patients with RAS/BRAF wild-type metastatic colorectal cancer (mCRC). Unfortunately, cetuximab resistance often occurs during targeted therapy. However, the underlying epigenetic mechanisms remain unclear. Our previous study demonstrated that the exosomal transfer of urothelial carcinoma-associated 1 (UCA1) confers cetuximab resistance to CRC cells. The goal of this study was to elucidate the detailed role of UCA1 in cetuximab resistance in CRC and the underlying molecular mechanism.

**Methods:** *In vitro* and *in vivo* functional studies were performed to assess the role of UCA1 in cetuximab resistance in CRC cell lines and xenograft models. Quantitative reverse transcription-polymerase chain reaction (qRT-PCR) was used to examine UCA1 localization and expression. Bioinformatics analysis was performed to predict the potential mechanism of UCA1, which was further validated by the dual-luciferase reporter assay and the RNA immunoprecipitation (RIP) assay. Cells treated with indicators were subjected to Cell Counting Kit-8 (CCK-8) and western blotting to investigate the role of hepatocyte growth factor (HGF)/c-mesenchymal-epithelial transition (c-MET) signalling in UCA1-mediated cetuximab resistance.

**Results:** We showed that UCA1 decreased CRC cell sensitivity to cetuximab by suppressing apoptosis. Mechanistic studies revealed that UCA1 promoted cetuximab resistance by competitively binding miR-495 to facilitate HGF and c-MET expression in CRC cells. Moreover, HGF was shown to attenuate the cetuximab-induced inhibition of cell proliferation by activating the HGF/c-MET pathway in CRC cells.

**Conclusion:** We provide the first evidence of a UCA1-miR-495-HGF/c-MET regulatory network involved in cetuximab resistance in CRC. Therefore, UCA1 has potential as a predictor and therapeutic target for cetuximab resistance.

## Introduction

Colorectal cancer (CRC) constitutes one of the leading causes of cancer-related morbidity and mortality worldwide 1. Cetuximab (CTX), an epidermal growth factor receptor (EGFR) monoclonal antibody (mAb), can negatively regulate tumour growth and induce an efficient antitumour effect. For the management of metastatic CRC (mCRC) patients, the integration of cetuximab and standard chemotherapy is a common therapeutic strategy that can improve the response rates and lower the risk of progression to mCRC 2. However, some RAS/BRAF wild-type patients acquire cetuximab resistance after the initial treatment period 3. Although some genomic alterations are associated with acquired resistance to cetuximab 4, for example, EGFR ectodomain mutation (S492R) can cause cetuximab resistance by preventing cetuximab binding, the underlying epigenetic mechanisms are still incomplete.

Long noncoding RNAs (lncRNAs) are generally defined as nonprotein coding transcripts > 200 nucleotides in length that play critical regulatory roles in chromatin modification, transcriptional regulation and posttranscriptional processing 5. LncRNAs are also considered key regulators in drug resistance. For example, ENST00000547547 influences the sensitivity of CRC cells to 5-fluorouracil (5-FU) by competitively inhibiting miR-31/ABCB9 6, and the LINC00152/miR-139-5p/NOTCH1 axis regulates chemoresistance by suppressing apoptosis 7. More recently, researchers further indicated the potential roles and the underlying mechanism of some lncRNAs in the field of acquired resistance to cetuximab 8-10, including lncRNA MIR100HG, POU5F1P4 and LINC00973, which may lead to the identification of novel therapeutic targets for treating late-stage CRC.

Studies over the last decade have revealed the critical role of extracellular vesicles (EVs) in cellular physiology, including the transfer of various cargoes and signals from donor cells to recipient cells, leading to the regulation of biological processes 11. Exosomes are small EVs that contain different materials, such as lipids, DNA, RNA and protein, and are critical mediators of intercellular communication 12. Exosomes play significant roles in cancer progression and metastasis, drug resistance and immune modulation 13. We previously demonstrated that exosomes from cetuximab-resistant CRC cells could alter the expression of lncRNA urothelial carcinoma-associated 1 (UCA1), a rising star among oncogenic lncRNAs, and induce cetuximab resistance in cetuximab-sensitive cells 14. UCA1-containing exosomes may predict cetuximab therapy efficacy in CRC patients. Although the role of UCA1 in tumorigenesis and resistance to some anticancer drugs has been extensively investigated in a number of previous studies, the function of UCA1 in cetuximab resistance in CRC and the associated regulatory mechanism have not been fully elucidated.

The present study demonstrated that UCA1 overexpression can increase the cetuximab resistance of CRC both *in vitro* and *in vivo*. Mechanistically, UCA1 was proven to promote cetuximab resistance in CRC by binding and inhibiting miR-495 to facilitate hepatocyte growth factor (HGF) and c-mesenchymal-epithelial transition (c-MET) expression, which leads to activation of the HGF/c-MET signalling pathway. Our results demonstrate critical roles for UCA1 in cetuximab resistance and thus have therapeutic implications for CRC.

## Materials and Methods

### Cell lines and clinical samples

Cetuximab-sensitive Caco2 cells (Caco2-CS) and cetuximab-resistant Caco2 cells (Caco2-CR) were previously described 14. Cetuximab was purchased from MERK (Darmstadt, Germany). Caco2-CS cells were cultured in basic medium (Dulbecco's modified Eagle's medium, DMEM) supplemented with 10% foetal bovine serum and 1% penicillin-streptomycin (Invitrogen, USA), and the complete medium used for culturing Caco2-CR cells contained cetuximab (3 μg/ml, Merck). Both cell lines were incubated at 37 °C in a humidified atmosphere containing 5% CO_2_ and 95% air. Cells were routinely tested for mycoplasma contamination every 2-3 months.

All human CRC samples were obtained from Harbin Medical University Cancer Hospital. The study was approved by the Ethics Committee of Harbin Medical University Cancer Hospital. We analysed 10 pairs of eligible tumour specimens before and after treatment with cetuximab. The main inclusion criteria were as follows: (1) specimens collected before cetuximab treatment or combined with chemotherapy during colonoscopy, biopsy or palliative operation; (2) pathologically confirmed mCRC; (3) wild-type KRAS and NRAS on codons 2, 3 and 4 and wild-type BRAF on codons 11 and 15 via targeted Sanger sequencing of the tumour tissue DNA; (4) an Eastern Cooperative Oncology Group (ECOG) performance status of 0 or 1; (5) written informed consent for publication of their clinical details from all subjects; and (6) computed tomography (CT) scans were performed and reviewed every 4 to 6 weeks to evaluate the clinical response using the Response Evaluation Criteria in Solid Tumours 1.1 guidelines 15. Clinical data, including response evaluation during the study, were collected. (7) When disease progression was indicated, posttreatment specimens were obtained at the time of progression. The specimens were immediately frozen in liquid nitrogen when removed and stored at -80 °C until DNA/RNA extraction. Tissue acquisition and handling of human tissue specimens were performed according to institutional and state regulations. The sequences of the oligonucleotide primers used for PCR are listed in Supplementary Table 1. The major clinicopathological characteristics of these patients are summarized in Supplementary Table 2.

### Vector constructs and chemicals

We amplified UCA1 from the cDNA of Caco2-CS cells using PrimerSTAR premix (TaKaRa, Japan) and subcloned it into the pLVX-Puro lentivirus plasmid, yielding pLVX-UCA1. A lentiviral vector packaging system (psPAX2-pMD2G) was cotransfected into HEK-293T cells to produce the desired lentivirus. Lentiviruses were collected after 48 h, and Caco2 cells were then infected in the presence of 5 μg/ml polybrene (Sigma-Aldrich, MO, USA). The infectious medium was aspirated and replaced with fresh complete medium after 24 h. Following puromycin selection for at least 2 weeks, stable transfection was finished, and Caco2-UCA1 cells overexpressing UCA1 and Caco2-NC cells (negative control) were obtained. UCA1 expression in Caco2-UCA1 and Caco2-NC cells was determined by quantitative reverse transcription-polymerase chain reaction (RT-qPCR).

Small interfering RNAs (siRNAs) for UCA1 and c-MET were chemically synthesized by GenePharma (Shanghai, China). Human HGF and HGF-neutralizing antibodies were obtained from R&D Systems (Minneapolis, MN). PHA-665752 (a highly specific c-MET inhibitor) was obtained from Santa Cruz Biotechnology (Santa Cruz, CA).

### RNA extraction and RT-qPCR analysis

Total RNA was extracted from cells or tissue specimens using TRIzol reagent (Invitrogen, Carlsbad, CA, USA). cDNA from one microgram of total RNA was obtained using a RevertAid RT Reverse Transcription Kit (Thermo Fisher Scientific, MA, USA). RT-qPCR was performed on a CFX96™ Real-Time PCR detection system (Bio-Rad) using PowerUp™ SYBR™ Green Master Mix (Thermo Fisher Scientific, MA, USA). Relative RNA expression levels were calculated using the 2^-ΔΔCt^ method and normalized to β-actin 14. To quantify the mature miRNAs, stem-loop RT-PCR assays (Applied Biosystems, USA) were performed, and U6 small nuclear RNA (snRNA) served as an internal control. The primer sequences are summarized in Supplementary Table 1.

### Cell viability assay

Exponentially growing cells (3 × 10^3^ cells/well) were seeded in 96-well plates and incubated overnight, after which cells were incubated with graded concentrations (0-800 μg/ml) of cetuximab and cultured for 48 h. Subsequently, Cell Counting Kit-8 (CCK-8; Bimake, Shanghai, China) reagent was dropped into each well and incubated at 37 °C for 1-3 h, after which the absorbance at 450 nm was determined using a microplate reader and recorded 16. The dose for 50% inhibition of cell metabolic activity (IC50) was generated from logarithmic sigmoidal dose-response curves via GraphPad Prism v6 software (GraphPad Inc.).

### Flow cytometric analysis

Cells were plated into T25 culture flasks (1.5 × 10^5^ cells) and cultured in 4 ml of complete medium for 24 h, after which the cells were treated with or without cetuximab (200 μg/ml) and incubated for 48 h. Cells were stained with a combination of Annexin V-APC and 7-amino-actinomycin D (7-AAD) and then analysed by flow cytometry (BD Biosciences, NJ, USA). For the analysis of cell cycle distribution, the cells were collected, incubated in cold 75% ethanol at 4 °C overnight, pelleted, stained with propidium iodide (PI)/RNase Staining Solution (BD Biosciences, NJ, USA) for 20 min, and subjected to flow cytometry 17.

### *In vivo* tumour growth in a xenograft model

Six- to 8-week-old female BALB/c athymic nude mice were obtained from the Experimental Animal Center of Harbin Medical University and kept under specific pathogen-free conditions with regulated day-night cycles. Animal studies were approved by the Harbin Medical University Animal Care Committee. Briefly, cell suspensions were subcutaneously injected into the flanks of nude mice (5 × 10^6^ tumour cells/150 μl PBS per spot). Tumour size was assessed by a bilateral calliper. Tumour volume (TV) calculations were obtained by using the formula TV = (L ×W^2^)/2, where L represents the tumour maximum diameter and W the right angle diameter to that axis 18. Once the tumour size reached approximately 100 mm^3^, the tested mice were then randomly assigned to the control saline (CTL) and cetuximab treatment groups. All mice were sacrificed following the national and institutional guidelines after one month of cetuximab treatment, and then the tumour size and weight were measured for each tumour, after which the tumours were formalin-fixed paraffin-embedded for haematoxylin and eosin (H&E) staining.

### Localization analysis

Cytoplasmic and nuclear fractions were prepared using an Ambion PARIS Kit (AM1921, Life Technologies) 19. In brief, Caco2 cells were washed with PBS, harvested, and then suspended in ice-cold fractionation buffer, after which the cytoplasmic and nuclear fractions were aspirated. The nuclear fraction was then incubated with lysis buffer. After the addition of ethanol and filtering applications through a cartridge, RNA was obtained from cell lysates. Then, 200 ng of cytoplasmic/nuclear RNA was reverse transcribed into cDNA and analysed by RT-qPCR; β-actin served as the control for the normalization of different genes and UCA1 expression.

### Dual-luciferase reporter assay

The 3'-untranslated regions (3'UTRs) of HGF and c-MET and the full-length UCA1 sequence were cloned into the pMir-Glo vector (Promega). Then, site-directed mutagenesis of the miR-495 binding sites within the UCA1 sequence and the HGF and c-MET 3'UTRs was performed to create mutant UCA1 and mutant 3'UTRs of HGF and c-MET. HEK-293T cells were seeded in 96-well plates and then cotransfected with miR-495 mimics (50 nM) or negative control RNA (miR-NC), luciferase reporter vector (50 ng), and pRL-CMV (5 ng) using Lipofectamine 3000 reagent. The luciferase activity was measured with the Dual-Luciferase Reporter Assay System (Promega, Madison, Wisconsin, USA) 48 h after transfection 20.

### RNA immunoprecipitation (RIP) assay

We performed RIP using an EZ-Magna RIP Kit (Millipore, Billerica, MA, USA) 21. Caco-2 cells grown to 70-80% confluency were scraped off and lysed in complete RIPA lysis buffer. Next, the cell extract was incubated in RIP buffer containing magnetic beads labelled with a human anti-Ago2 antibody or negative control mouse IgG (Millipore). Samples were incubated with proteinase K with shaking to digest, and the immunoprecipitants were isolated. Finally, purified RNA was analysed by RT-qPCR.

### Transient transfection

We performed transient transfection with miR-495 mimics or miR-NC (GenePharma, Shanghai, China) using Lipofectamine 3000 from Invitrogen 22.

### Measurement of the HGF concentration in the cell culture medium

The HGF levels in the culture media of Caco2-UCA1 and Caco2-NC cells were measured by enzyme-linked immunosorbent assay (ELISA) using an HGF Human ELISA Kit from Invitrogen (KAC2211) 23. A standard curve was established using recombinant HGF.

### Western blotting (WB)

Caco-2 cells were homogenized in Mammalian Cell Lysis/Extraction Reagent (Sigma, St. Louis, MO) supplemented with 1% protease inhibitor cocktail and 1% Triton X-100 (TX-100), and lysates were obtained. Protein concentrations were measured using a BCA Protein Assay Kit (Sigma-Aldrich, St. Louis, MO, USA). Equal amounts of protein lysate (35 micrograms) were subjected to sodium dodecyl sulfate-polyacrylamide gel electrophoresis (SDS-PAGE) and transferred onto PVDF membranes for WB analysis with the primary antibodies and corresponding secondary antibodies (Supplementary Table 3). An ECL Western blotting kit from Beyotime Biotechnology was used for the detection of proteins. Subsequently, the bands on X-ray films were scanned and analysed.

### Statistical analysis

All statistical analyses were performed using the Statistical Package for the Social Science (SPSS) version 24 (Chicago, IL, USA). Data are presented as the means ± standard deviation (SD) from three separate experiments. Measurement data were compared using Student's t-test and one- or two-way ANOVA. The correlations between the expression of UCA1, miR-495 and HGF or c-MET were determined by Spearman correlation analysis. All *p* values were two-sided and indicated significance if less than 0.05.

## Results

### UCA1 promotes cetuximab resistance *in vitro* and *in vivo*

**Figure 1:**
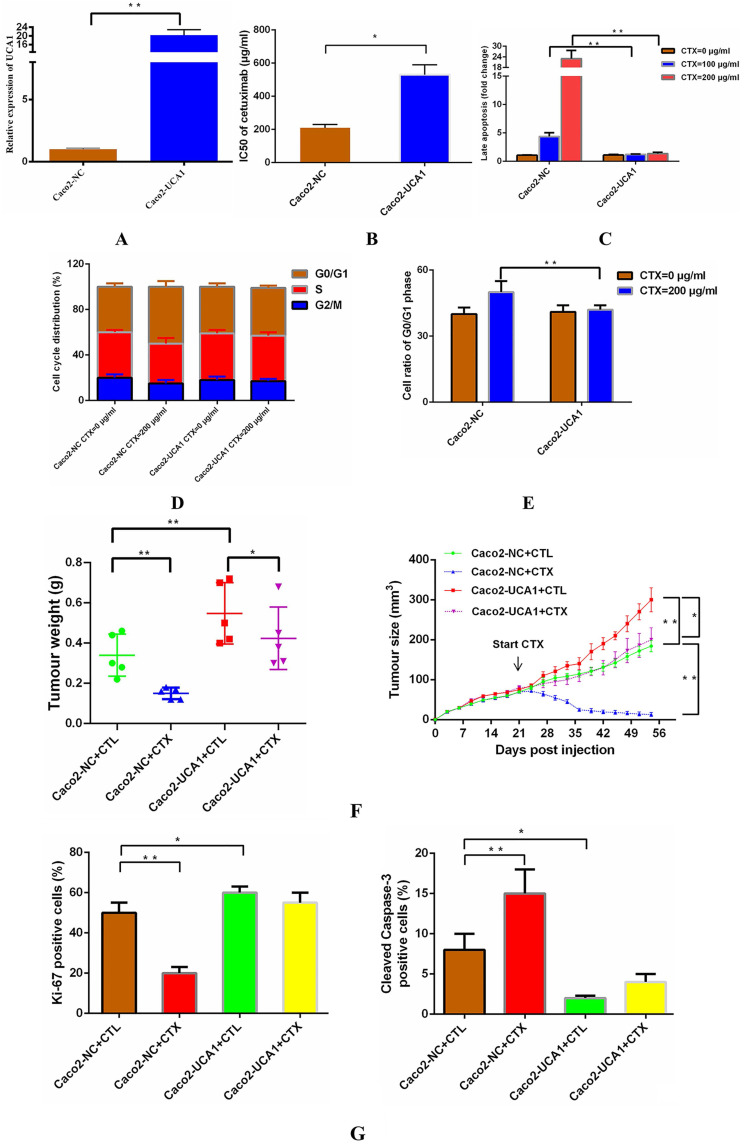
**UCA1 promotes cetuximab resistance *in vitro* and *in vivo*. (A)** UCA1 expression was dramatically upregulated in Caco2-UCA1 cells. **(B)** The IC50 values of cetuximab were significantly higher in Caco2-UCA1 cells than in Caco2-NC cells. **(C)** The percentage of late apoptotic cells was significantly higher in Caco2-NC cells than in Caco2-UCA1 cells. **(D-E)** UCA1 overexpression did not cause significant changes in the cell cycle distribution, while G0/G1 phase cell cycle arrest was inhibited by cetuximab. **(F)** UCA1 affects cetuximab resistance *in vivo*. **(G)** Xenografts from the Caco2-NC group demonstrated less Ki-67 staining (left) and increased cleaved caspase-3 staining (right) than those observed in the control group after cetuximab treatment. *P < 0.05, **P < 0.01.

We delivered a lentiviral-based UCA1 overexpression construct or an empty vector into Caco2 cells to generate the stable cell lines Caco2-UCA1 and Caco2-NC, respectively. UCA1 expression was examined by RT-qPCR, and UCA1 was significantly upregulated in Caco2-UCA1 cells compared with Caco2-NC cells (Fig. 1A). To assess the level of cetuximab resistance in CRC cells, Caco2-NC and Caco2-UCA1 cells were challenged with cetuximab, and the IC50 value was calculated. UCA1 upregulation in Caco2-UCA1 cells increased the IC50 of cetuximab, as measured by CCK-8 assay (Fig. 1B). For cell apoptosis analysis, Caco2-NC cells had an increased percentage of late apoptotic cells compared to Caco2-UCA1 cells after 48 h of cetuximab treatment (Fig. 1C). Interestingly, although UCA1 overexpression did not cause significant changes in the distribution of the cell cycle (Fig. 1D), cell cycle arrest at G0/G1 phase was inhibited after cetuximab treatment (Fig. 1E). Together, our results demonstrate that UCA1 induces resistance to cetuximab in CRC cells.

To determine whether UCA1 affects cetuximab resistance *in vivo*, we subcutaneously injected Caco2-NC or Caco2-UCA1 cells into athymic nude mice. Once the mean tumour volume reached ~100 mm^3^, cetuximab or CTL was injected intraperitoneally. The tumour regressed, and the tumour weight was significantly decreased upon administration of cetuximab in the mice implanted with Caco2-NC or Caco2-UCA1 cells, although the tumours in the mice implanted with Caco2-UCA1 cells continued to grow after cetuximab treatment (Fig. 1F). We observed that the Caco2-NC group xenografts presented less IHC Ki-67 staining and increased IHC cleaved caspase-3 staining than those in the control group in the presence of cetuximab (Fig. 1G). Our data suggest that increased UCA1 expression promotes cetuximab resistance *in vivo*.

### UCA1 functions as a miR-495 sponge

**Figure 2:**
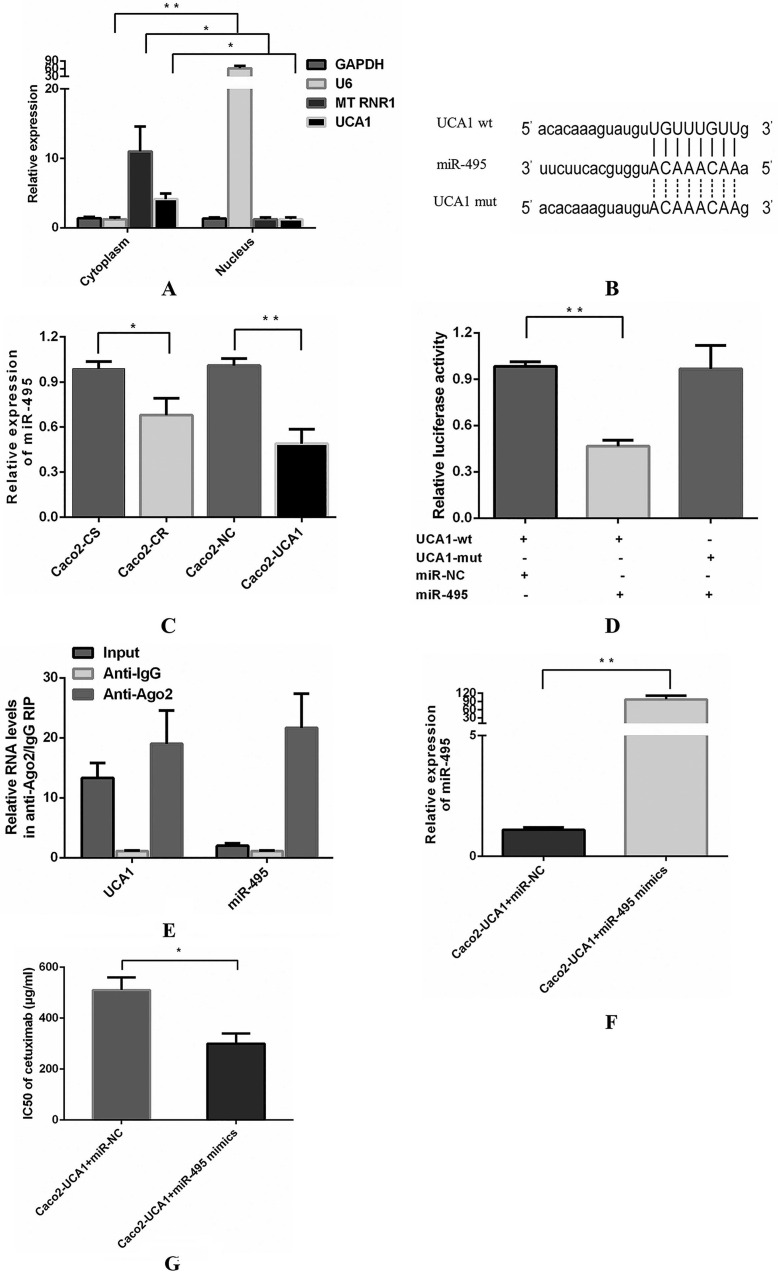
** UCA1 functions as a miR-495 sponge. (A)** UCA1 is primarily expressed in the cytoplasm. **(B)** Schematic comparison between UCA1 and the “seed sequence” of miR-495. **(C)** The miR-495 level was lower in not only Caco2-CR cells than in Caco2-CS cells but also in Caco2-UCA1 cells than in Caco2-NC cells. U6 served as an internal reference for normalization. **(D)** The luciferase activity of cells cotransfected with wild-type UCA1 and miR-495 mimics was significantly reduced compared with those cotransfected with wild-type UCA1 and miR-NC, while this repression of luciferase activity was completely refractory, after which cells were transfected with the UCA1 mutated vector. **(E)** Compared to the control immunoprecipitants, UCA1 and miR-495 were found to be preferentially enriched in Ago2-containing miRNPs. **(F)** Upregulated expression of miR-495 was found in Caco2-UCA1 cells transfected with miR-495 mimics compared with the mimic control group. U6 served as an internal reference for normalization. **(G)** miR-495 upregulation in Caco2-UCA1 cells decreased the IC50 of cetuximab. *P < 0.05, **P < 0.01.

Then, we explored the subcellular localization of UCA1 using RT-qPCR to reveal the underlying mechanism by which UCA1 functions. RNA from cytoplasmic and nuclear lysates of Caco2 cells was converted into cDNA and then analysed by RT-qPCR to determine the levels of UCA1. We assessed the levels of three different genes as positive controls, including GAPDH, which is present in both the cytoplasm and nucleus, U6 (nuclear control) and the mitochondrial gene MT RNR1 (cytoplasmic control). UCA1 was found primarily in the cytoplasm (Fig. 2A), indicating that UCA1 may serve as an important regulator of gene expression at the posttranscriptional level. Researchers have shown that UCA1 may act as a competing endogenous RNA (ceRNA) to participate in the regulation of target gene expression in the cytoplasm 24-26. It is hypothesized that UCA1 may contribute to enhanced cetuximab resistance by inhibiting miRNA. A potential miR-495 binding site was identified in the UCA1 sequence using bioinformatics analyses (TargetScan, RNAhybrid and miRanda) (Fig. 2B). Interestingly, miR-495 was previously shown to increase sensitivity to cisplatin resistance, multidrug resistance (MDR) and platinum resistance in several cancers 27-29. Therefore, we assessed the potential association between UCA1 and miR-495 and observed that the miR-495 level was not only lower in Caco2-CR cells than in Caco2-CS cells but also lower in Caco2-UCA1 cells than in Caco2-NC cells (Fig. 2C). The dual-luciferase reporter assay revealed that the luciferase activity of cells cotransfected with wild-type UCA1 and miR-495 mimics was significantly reduced compared with those cotransfected with wild-type UCA1 and miR-NC (Fig. 2D). Interestingly, miR-495-mediated repression of luciferase activity was completely refractory to the cells transfected with the mutated vector (pmirGLO-UCA1-mut) (Fig. 2D), which has the mutated binding site for miR-495 in UCA1 (Fig. 2B). These results indicate that there is a sequence-specific interaction between miR-495 and UCA1.

Next, we performed RIP assays to examine the association between UCA1 and miR-495 in the form of miRNA ribonucleoprotein complexes (miRNPs). The RNA levels in immunoprecipitants were determined by RT-qPCR. Compared to the control immunoprecipitants, UCA1 and miR-495 were preferentially enriched in Ago2-containing miRNPs (Fig. 2E), suggesting an association between UCA1 and miR-495 in Ago2-containing miRNPs. In summary, UCA1 directly bound to miR-495 and negatively regulated its expression by acting as a sponge for miRNA. Subsequently, we investigated the effect of miR-495 on UCA1-induced resistance to cetuximab. miR-495 expression was evaluated by RT-qPCR at 48 h after miR-NC or miR-495 mimic transfection (Fig. 2F). miR-495 upregulation in Caco2-UCA1 cells decreased the IC50 of cetuximab (Fig. 2G). In summary, UCA1 induces cetuximab resistance in CRC cells by inhibiting miR-495 expression.

### UCA1 modulates HGF and c-MET expression in a miR-495-dependent manner

**Figure 3:**
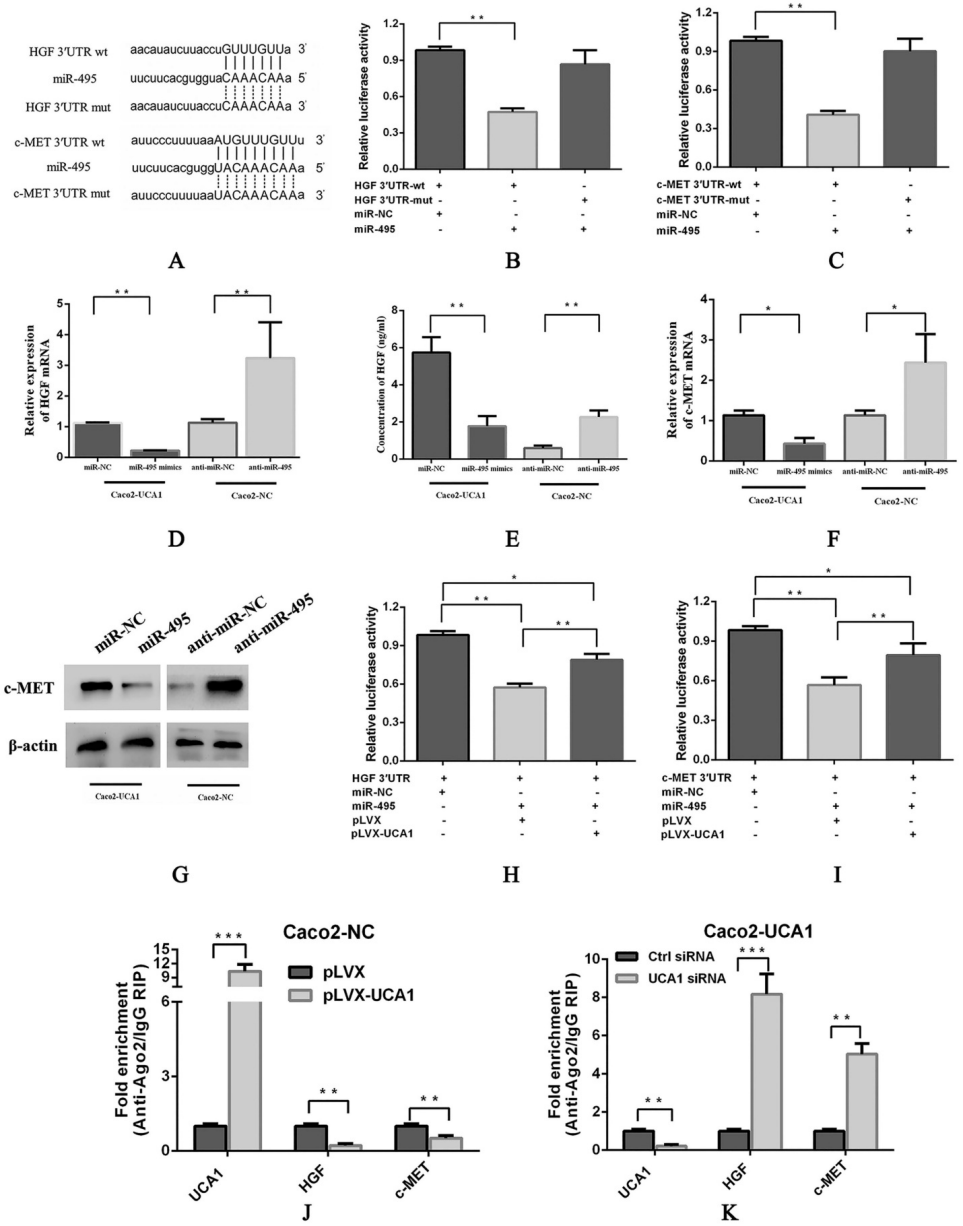
** UCA1 modulates HGF and c-MET expression in a miR-495-dependent manner. (A)** Schematic comparison between HGF (top) or c-MET (bottom) and the “seed sequence” of miR-495. miR-495 significantly inhibited the luciferase activity of the pmirGLO-HGF 3'UTR-wt **(B)** and pmirGLO-c-MET 3'UTR-wt **(C)** reporters but not that of the pmirGLO-3'UTR-mut reporter. miR-495 overexpression in Caco2-UCA1 cells decreased the mRNA expression **(D)** and secreted protein levels of HGF **(E)** and the mRNA expression **(F)** and protein levels of c-MET **(G)**, while miR-495 knockdown increased HGF (D) and c-MET mRNA (F) and protein levels (E, G) in Caco2-NC cells. β-actin was used as an internal control. In the presence of UCA1, the expression of reporter genes was restored in the pmirGLO-HGF 3'UTR **(H)** or pmirGLO-c-MET 3'UTR group **(I)**. **(J)** UCA1 overexpression led to enriched levels of Ago2 on UCA1 transcripts but substantially decreased those on HGF and c-MET transcripts. **(K)** UCA1 knockdown induced a significant increase in Ago2 recruitment to HGF and c-MET transcripts. *P < 0.05, **P < 0.01, ***P < 0.0001.

To further understand the possible mechanism of miR-495, we assessed several databases, including TargetScan, RNA22, PITA, PicTar and miRanda, to identify specific targets of miR-495 in the EGFR signalling pathway. Among the candidate miR-495 targets, HGF and c-MET were both observed to harbour miR-495 binding sites in their 3'UTRs in 4 databases (Fig. 3A). Interestingly, previous reports have shown that SRI 31215, an endogenous inhibitor of HGF activation, inhibits fibroblast-induced c-MET activation, epithelial-mesenchymal transition and migration of DU145 cells. Importantly, SRI 31215 treatment can overcome primary resistance to EGFR inhibitors in HGF-producing colon cancer cells and prevent fibroblast-mediated resistance to EGFR inhibitors 30. Therefore, we paid close attention to HGF and c-MET in subsequent investigations. pmirGLO-HGF 3'UTR-wt or pmirGLO-c-MET 3'UTR-wt, which contains wild-type 3'UTRs of HGF or c-MET, respectively, pmirGLO-HGF 3'UTR-mut or pmirGLO-c-MET 3'UTR-mut, which contains mutant 3'UTRs of HGF or c-MET, respectively, were cotransfected with either miR-NC or miR-495 into HEK293T cells, luciferase activity was significantly inhibited in the miR-495 and 3'UTR-wt cotransfection group (Fig. 3B and 3C, respectively), but not in the miR-495 and 3'UTR-mut cotransfection group, indicating that HGF and c-MET are target genes of miR-495. In addition, the mRNA expression (Fig. 3D) and secreted protein levels of HGF (Fig. 3E) and the mRNA expression (Fig. 3F) and protein levels of c-MET (Fig. 3G) were decreased after miR-495 overexpression in Caco2-UCA1 cells, while miR-495 knockdown increased the mRNA expression of HGF (Fig. 3D) and c-MET (Fig. 3F) and the c-MET protein levels (Fig. 3G) in Caco2-NC cells. In summary, by combining bioinformatics analyses with experimental verification, we revealed that miR-495 directly targets HGF and c-MET and regulates their expression in CRC.

Furthermore, we performed luciferase assays to evaluate the interaction among UCA1, miR-495 and HGF. Reporter gene expression was restored in the pmirGLO-HGF 3'UTR (Fig. 3H) and pmirGLO-c-MET 3'UTR (Fig. 3I) groups in the presence of UCA1, suggesting that UCA1 functions as a sponge by directly binding miR-495 to abolish the miRNA-mediated repression of the HGF and c-MET 3'UTRs. Moreover, UCA1 overexpression in Caco2 cells led to enriched levels of Ago2 on UCA1 transcripts but substantially decreased those on HGF and c-MET transcripts (Fig. 3J). In addition, UCA1 knockdown in Caco2-UCA1 cells induced a significant increase in Ago2 recruitment to HGF and c-MET transcripts compared to that observed in control cells (Fig. 3K). These results demonstrate that UCA1 upregulates HGF and c-MET expression in a miR-495-dependent manner.

### UCA1-mediated HGF expression rescues CRC cells from cetuximab inhibition through c-MET

**Figure 4:**
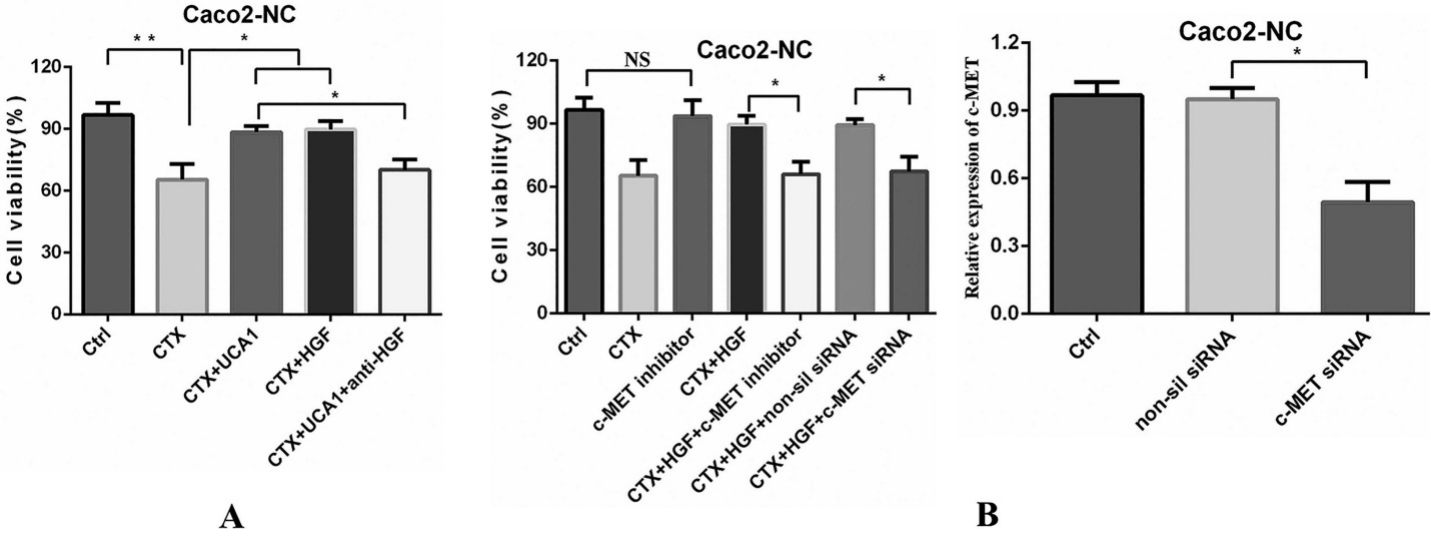
** UCA1-mediated HGF-dependent MET activation rescues Caco2-NC cells from cetuximab inhibition. (A)** Cetuximab (200 µg/ml) decreased cell viability, whereas cell proliferation significantly increased after the combination of cetuximab (200 µg/ml) with pLVX-UCA1 or HGF (10 µg/ml) treatment compared to cetuximab alone treatment. Treatment with an HGF neutralizing antibody (30 µg/ml) significantly hampered cell proliferation caused by UCA1 overexpression. **(B)** Left panel: Caco2-NC cells were treated with a combination of cetuximab (200 µg/ml) and HGF (10 µg/ml) with or without PHA-665752 (0.4 µM), and PHA-665752 completely abrogated the rescue effect of HGF and essentially restored the cetuximab-mediated inhibitory effects. SiRNA-mediated c-MET expression downregulation (right panel) abolished HGF-induced cetuximab resistance. NS, not significant. *P < 0.05, **P < 0.01.

Next, we examined the influence of UCA1 overexpression-mediated HGF expression on cetuximab resistance. The cell viability was reduced by approximately 40% after cetuximab treatment, whereas the cell proliferation significantly increased after the combination of cetuximab with pLVX-UCA1 or HGF treatment compared to cetuximab alone treatment (Fig. 4A). In contrast, treatment with a human HGF neutralizing antibody significantly attenuated UCA1 overexpression-induced Caco2-NC cell proliferation (Fig. 4A). Taken together, our data indicate that UCA1-mediated HGF expression can rescue CRC cells from cetuximab inhibition.

The HGF/c-MET pathway has been shown to mediate vascular endothelial growth factor receptor (VEGFR) inhibitor resistance and vascular remodelling in non-small-cell lung cancer (NSCLC) 31. Therefore, we next tested whether the rescue effect of HGF could be abrogated by inhibiting c-MET. A small molecule ATP-competitive inhibitor of c-MET, PHA-665752, had no significant effect on cell proliferation (Fig. 4B). However, when cells were treated with a combination of HGF and cetuximab with or without PHA-665752, PHA-665752 completely abrogated the rescue effect of HGF and restored the cetuximab-mediated inhibitory effects on the Caco2-NC cell line (Fig. 4B). We confirmed these results by using the RNA interference (RNAi) approach. SiRNA-induced c-MET downregulation abolished the HGF-induced cetuximab resistance of Caco2-NC cells (Fig. 4B), indicating that UCA1-mediated HGF expression induces cetuximab resistance through c-MET.

### Rescue of UCA1-mediated HGF expression occurs through c-MET-dependent AKT and MAPK activation

**Figure 5:**
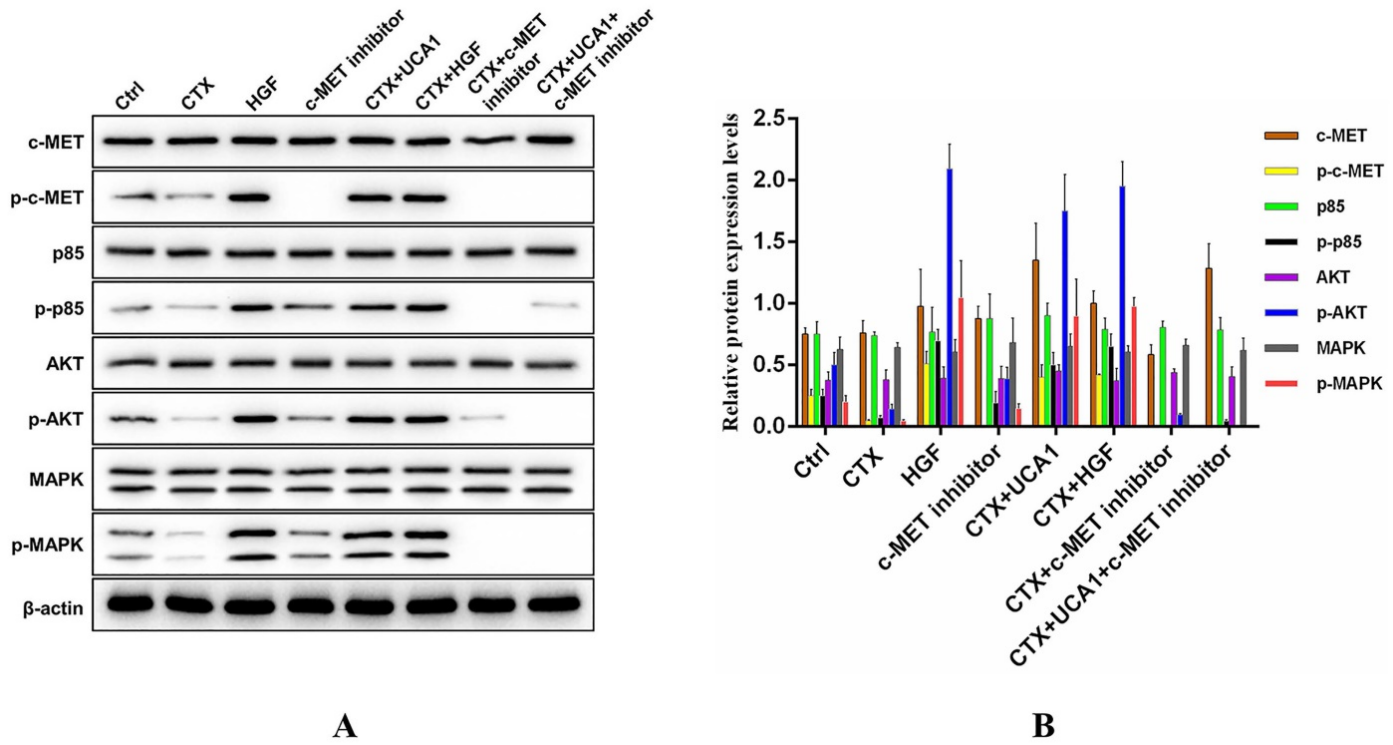
** Western blotting analysis of c-MET/PI3K/AKT/MAPK activation in Caco2-NC cells.** (A) Cetuximab significantly reduced the phosphorylated protein levels of c-MET, PI3K p85, AKT and MAPK. UCA1 and HGF restored the phosphorylated protein levels of c-MET, PI3K p85, AKT and MAPK to some extent, and the UCA1-mediated effects were abrogated by PHA-665752 treatment. β-actin served as a loading control. Images are representative of three independent experiments. (B) Quantitative analysis of Western blotting.

To further investigate the underlying mechanism by which UCA1-mediated HGF expression rescues the growth-inhibiting effects of cetuximab on CRC cells, we measured the activation status of downstream signalling molecules. Caco2-NC cells were treated as indicated, and the impacts of the PI3K/AKT and RAS/MAPK signalling pathways were assessed by Western blot to detect the phosphorylated and total levels of proteins in these pathways. Phosphorylated protein levels of c-MET, PI3K p85, AKT and MAPK were significantly decreased after cetuximab treatment, while cetuximab treatment did not affect the total protein levels of c-MET, PI3K p85, AKT and MAPK (Fig. 5A). In addition, UCA1 and HGF restored the phosphorylated protein levels of c-MET, PI3K p85, AKT and MAPK to some extent (Fig. 5A), and the effects of UCA1 were abrogated by treatment with PHA-665752 (Fig. 5A). Fig. 5B demonstrates the Western blotting quantitative data for the proteins of interest. Our results suggested that UCA1-mediated HGF expression rescued the growth-inhibiting effects of cetuximab through activation of the c-MET/PI3K/AKT/MAPK signalling pathway in CRC cells.

### UCA1-miR-495-HGF/c-MET axis correlation analysis of paired samples collected before treatment and after acquired resistance

**Figure 6:**
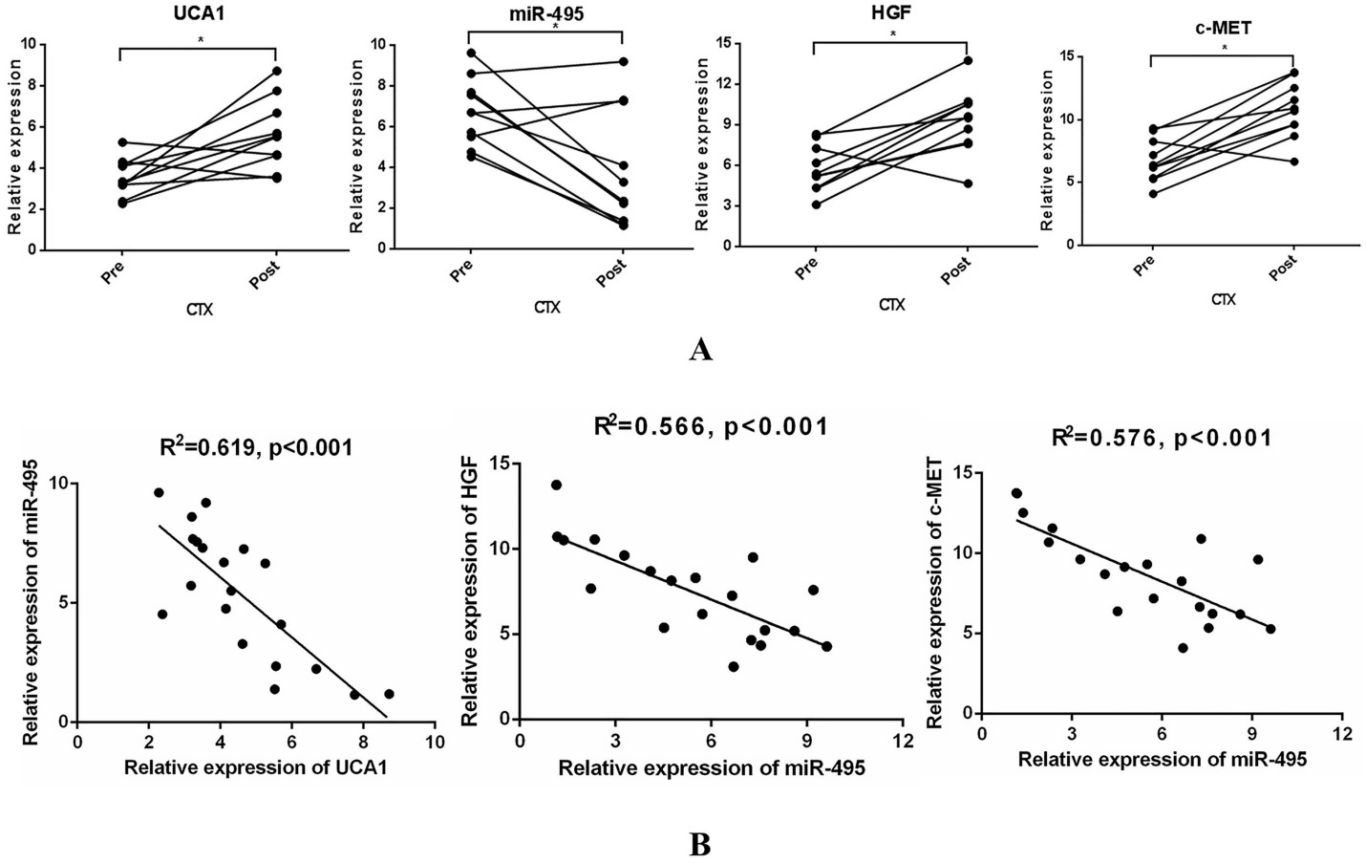
** Specimens from CRC patients on cetuximab exhibit increased UCA1, HGF, and c-MET levels and decreased miR-495 levels at the time of progression. (A)** UCA1, HGF and c-MET expression was increased, while miR-495 expression was decreased after resistance to cetuximab occurred. **(B)** A significant inverse correlation between miR-495 expression and UCA1 (left) or HGF (middle) or c-MET (right) expression was observed. *P < 0.05.

To examine whether UCA1-miR-495-HGF/c-MET axis-mediated resistance to cetuximab is of great significance in the clinic, 10 paired tumour specimens were obtained (Supplementary Table 2). UCA1, HGF and c-MET expression was increased, while miR-495 expression was decreased after resistance to cetuximab occurred (Fig. 6A). In addition, a significant inverse correlation was observed between UCA1 expression and that of miR-495 or miR-495 expression and that of HGF and c-MET (Fig. 6B). These clinical data supported our preclinical findings and demonstrated that the UCA1-miR-495-HGF/c-MET axis contributes to the development of acquired cetuximab resistance in patients with CRC.

## Discussion

In China, the incidence and mortality of CRC has risen rapidly in recent years 32. The administration of mAbs against EGFR, such as cetuximab and panitumumab, combined with conventional chemotherapy can prolong the survival of mCRC patients with wild-type RAS 33. Unfortunately, increasing evidence indicates that many patients who initially respond to cetuximab eventually acquire resistance. Advances in genomic technologies have led to the identification of a variety of genetic markers of resistance to anti-EGFR therapy 34. However, there are only a few reports on the connection between lncRNAs and cetuximab resistance. Although researchers established cetuximab-resistant cells in three-dimensional (3D) culture, they could not identify new genetic factors contributing to cetuximab resistance through sequencing analysis. The expression levels of the lncRNA MIR100HG and its derived miR-100 and miR-125b were shown to be significantly increased in cetuximab-resistant cells. In addition, miR-100 and miR-125b synergistically regulate several regulators of Wnt/β-catenin signalling, leading to the activation of Wnt signalling. Moreover, ICG-001, a β-catenin/CBP inhibitor, restored responsiveness to cetuximab *in vitro* and *in vivo* 8. Mining of the GEO database revealed the decreased expression of lncRNA POU5F1P4 in cetuximab-resistant cells and tissues and the association between lncRNA POU5F1P4 expression and mCRC patient survival. Downregulation of POU5F1P4 could decrease the sensitivity of CRC cells to cetuximab 9. In addition, Jing et al. found that several groups of lncRNAs might be involved in pathways associated with cetuximab resistance. Among them, LINC00973 expression is higher in three cetuximab-resistant CRC cell lines, and LINC00973 siRNA can ameliorate cetuximab resistance in SW480 cells 10.

UCA1 was initially considered an oncogenic lncRNA in bladder cancer and was revealed to be overexpressed in multiple cancers in subsequent studies 35. UCA1 primarily promotes tumorigenesis by binding to probable tumour-suppressive miRNAs, activating several key signalling pathways and altering transcriptional and epigenetic regulation 36. In addition, the pivotal role of UCA1 in the acquisition of resistance to anticancer drugs has been proven for several cancers. Overexpression of UCA1 enhances chemotherapy resistance, while knockdown of UCA1 increases sensitivity to drug treatment 37. We previously established a cetuximab-resistant CRC cell line (Caco2-CR) and demonstrated the potential application of exosomal UCA1 to predict the responses of CRC patients to cetuximab therapy. Moreover, exosomes of cetuximab-resistant CRC cells can transmit cetuximab resistance to sensitive cells by delivering UCA1 14. However, the function of UCA1 in cetuximab resistance in CRC and the underlying mechanism have remained unelucidated.

Several studies have shown that UCA1 is overexpressed in CRC cell lines and tissues. One study demonstrated that UCA1 amplification can promote cell proliferation and tumorigenicity by sponging miR-204-5p 38. Another study demonstrated that there was a significant association between UCA1 expression and tumour stage, lymphatic metastasis status and patient survival in CRC. Further functional experiments showed that silencing UCA1 suppressed cell proliferation and metastasis and induced G0/G1 growth arrest and apoptosis 39. These findings clearly suggest the oncogenic role of UCA1 in CRC. High UCA1 expression is also reported to be related to enhanced resistance to anticancer drug treatments, including cisplatin, doxorubicin, paclitaxel, tamoxifen and 5-FU 40. For example, Bian et al. demonstrated that UCA1 can sponge miR-204-5p and then regulate its target genes, thereby contributing to resistance to 5-FU 38. In this study, we found that UCA1 overexpression promoted cetuximab resistance, both *in vitro* and *in vivo*, confirming its role in anticancer drug resistance in CRC, which was consistent with previous similar findings 41.

According to their functions, lncRNAs can be roughly divided into four categories: decoy, signal, scaffold and guide 42. The function of lncRNAs is associated with their unique subcellular localization patterns 43. Cytoplasmic lncRNAs may interfere, for example, with protein or posttranslational modifications leading to altered signal transduction 44. Han et al. demonstrated that the lncRNA CRNDE was able to stimulate cell proliferation and chemoresistance by way of miR-181a-5p-mediated regulation of Wnt/β-catenin signalling in CRC 45. The lncRNA SNHG6 was reported to promote CRC cell growth, invasion, and migration by interacting with miR-26a, miR-26b, and miR-214 and their common target EZH2 46. Over 30 articles have investigated the ability of UCA1 to indirectly regulate processes by sequestering miRNAs and involving downstream targeted transcript degradation in the last 5 years. UCA1 functions by interacting with over 20 different miRNAs, and the expression levels of more than 30 different targeted genes are changed due to the interactions of UCA1 and miRNA 40. In combination with bioinformatics analysis and experimental method verification, we proved that UCA1 promotes cetuximab resistance by binding miR-495 and then inhibiting its expression in CRC. miR-495 is located at chromosome 14q32.31 in the human genome and has been extensively investigated. Along with other miRNAs in the 14q32.31 cluster, miR-495 is involved in the development of normal tissue and participates in cancer cell proliferation, metastasis, and apoptosis 47. MiR-495 and other miRNAs encoded by the 14q32.31 gene cluster may also increase chemosensitivity in lung, breast, and pancreatic cancers and leukaemia, which could improve survival. For example, miR-495 overexpression was shown to downregulate ATPase copper transporting α (ATP7A) and enhance cisplatin sensitivity in NSCLC. Additionally, miR-495 can increase the intracellular cisplatin concentration, and ATP7A overexpression was shown to reduce the effects of miR-495 on cell processes 29. In small cell lung cancer (SCLC), miR-495, miR-134 and miR-379, which are located in chromosomal region 14q32.31, have been demonstrated to increase sensitivity to anticancer drugs, including cisplatin, etoposide and doxorubicin 48. Similarly, miR-495 was shown to increase chemotherapeutic sensitivity in chronic myeloid leukaemia (CML) 49. In pancreatic cancer (PaCa), garcinol was observed to sensitize PaCa cells to gemcitabine treatment by modulating miR-495 and activating miR-495-associated signalling pathways 50. The role of miR-495 in tumours is controversial, as it has been shown to function as a tumour suppressor or an oncogene, but its ability to restore responsiveness to anticancer drugs is consistent with our findings. However, the rescue assay suggested that transfection of miR-495 mimics partially counteracted the resistance to cetuximab caused by UCA1 overexpression, indicating additive mechanisms involving UCA1-mediated cetuximab resistance.

The regulatory roles of miR-495 in disease are primarily determined by its negative effects on the expression of its target mRNAs. As cetuximab binds competitively and has high affinity for EGFR, we focused on the major EGFR activation-induced downstream signalling pathways, which are also involved in resistance to EGFR inhibitors 51. Using five miRNA target prediction algorithms, HGF and c-MET were identified as targets of miR-495 by one or more algorithms. In our present study, bioinformatics analysis and experimental method verification were used in combination to confirm that miR-495 directly targeted HGF and c-MET mRNA in CRC cells as well as in cetuximab-treated CRC patients whose tumours had progressed. Collectively, our data suggest that UCA1-mediated HGF expression can rescue CRC cells from cetuximab-mediated inhibition.

Epigenetic instability involves histone modification, aberrant DNA methylation, chromosome remodelling, and noncoding RNA interference 52, with miRNA interference and aberrant methylation phenotypes being closely associated with both CRC development and drug resistance 53. miRNAs are endogenous small noncoding RNAs that can regulate gene expression at the posttranscriptional level. Despite initial research on miRNA function in the area of anti-EGFR mAb resistance, its role cannot be ignored due to the underlying mechanisms depending on epigenetic aberrations 54. Researchers have reported that some miRNAs are involved in resistance to EGFR-targeted therapy in mCRC with relatively clear molecular mechanisms 8, 55-57. For example, miR-199a-5p and miR-375 negatively regulate the AKT pathway by targeting PHLPP1 55, whereas miR-100 and miR-125 coordinately activate Wnt signalling 8. c-MET is a receptor tyrosine kinase for HGF and is characterized by its ability to promote tumorigenesis 58. HGF/c-MET signalling induces cell proliferation, differentiation, migration, invasion, and EMT, promoting tumourigenesis and tumour progression 59. An increasing number of studies have demonstrated that the HGF/c-MET pathway may be a potential therapeutic target for the treatment of a variety of cancers, including CRC 60. The antitumour activity of c-MET inhibitors has been evaluated in the treatment of NSCLC in preclinical and clinical trials, and the combined use of EGFR-TKIs and c-MET inhibitors may be a promising treatment option for EGFR-TKI-naive and EGFR-TKI-resistant NSCLC patients 61. Thus, we focused on HGF/c-MET signalling in our present study. Here, siRNA-mediated c-MET downregulation or PHA-665752-mediated c-MET tyrosine kinase activity inhibition prevented HGF-dependent activation of the PI3K/AKT and MAPK pathways and resensitized cells to cetuximab-induced growth-inhibitory effects. Many c-MET-targeting therapeutics, including TKIs, mAbs, and molecular decoys, are currently undergoing clinical development 62-65. The efficacy and safety of combined receptor tyrosine kinase (RTK) therapy should be evaluated *in vivo* to overcome EGFR TKI resistance in the future. In addition, the inability of murine HGF to bind and effectively activate human c-MET should also be considered thoroughly to overcome the inherent inadequacies 66. Elucidation of the mechanism underlying the UCA1-miR-495-HGF/c-MET regulatory network involved in cetuximab resistance will lead to the development of novel agents and therapeutic approaches for CRC in the future.

**Figure 7:**
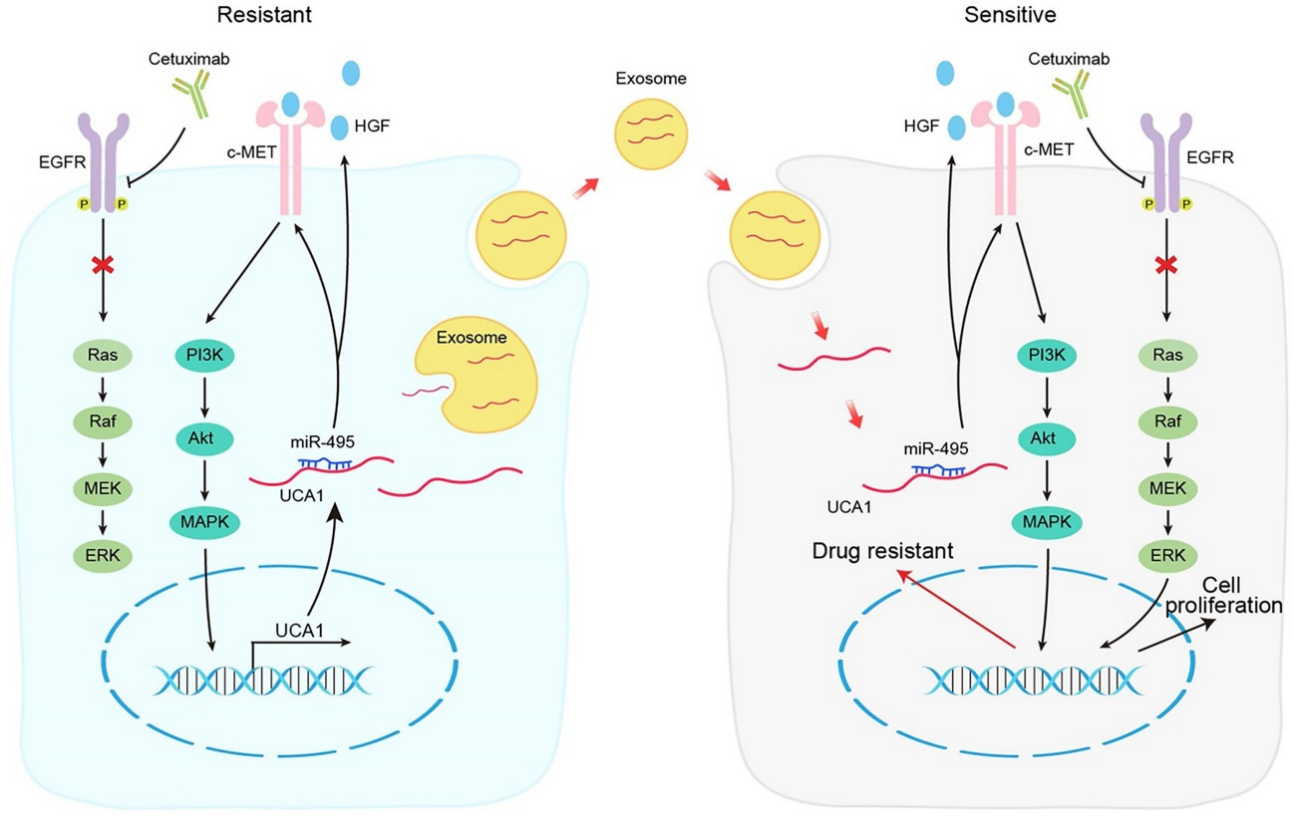
Schematic diagram of the mechanism of UCA1 in cetuximab resistance.

In conclusion, our results demonstrate that the UCA1-miR-495-HGF/c-MET regulatory network represents a novel cetuximab resistance mechanism in CRC (Fig. 7). Our findings add to the increasing evidence suggesting the use of combinatorial treatments to mitigate or overcome resistance to therapeutic drugs by targeting a single signalling pathway in patients with cancer.

## Supplementary Material

Supplementary tables.

## Abbreviations

CRC: colorectal cancer
UCA1: urothelial carcinoma-associated 1
HGF: hepatocyte growth factor
c-MET: c-mesenchymal-epithelial transition
CTX: cetuximab
EGFR: epidermal growth factor receptor
mAb: monoclonal antibody
mCRC: metastatic colorectal cancer
lncRNAs: long noncoding RNAs
5-FU: 5-fluorouracil
EVs: extracellular vesicles
DMEM: Dulbecco's modified Eagle's medium
FBS: foetal bovine serum
CT: computed tomography
RECIST: Response Evaluation Criteria in Solid Tumours
qRT-PCR: quantitative reverse transcription-polymerase chain reaction
siRNAs: small interfering RNAs
snRNA: small nuclear RNA
CCK-8: Cell Counting Kit-8
7-AAD: 7-amino-actinomycin D
PI: propidium iodide
H&E: haematoxylin and eosin
3'UTRs: 3'-untranslated regions
RIP: RNA immunoprecipitation
IgG: immunoglobulin G
ELISA: enzyme-linked immunosorbent assay
SD: standard deviation
qPCR: quantitative real-time PCR
ceRNA: competing endogenous RNA
MDR: multidrug resistance
miRNPs: miRNA ribonucleoprotein complexes
VEGFR: vascular endothelial growth factor receptor
NSCLC: non-small-cell lung cancer
TKIs: tyrosine kinase inhibitors
ATP7A: ATPase copper transporting α
SCLC: small cell lung cancer
CML: chronic myeloid leukaemia
RTK: receptor tyrosine kinase
