# Supplementary material for: LncRNA UCA1 mediates Cetuximab resistance in Colorectal Cancer via the MiR-495 and HGF/c-MET Pathways
Source: J Cancer. 2022 Jan 1;13(1):253–67. doi: 10.7150/jca.65687 (PMC8692674; doi:10.7150/jca.65687)
Supplement: Supplementary file 1 — Supplementary tables. [file jcav13p0253s1.pdf]

Supplementary Table 1. Detailed information on the primer sequences

**Primers for real time PCR**	
UCA1-F	AAGAGATCCACCTGCGACCT
UCA1-R	CCTCCACCGTAAGAGTTACC
GAPDH-F	CATGGCCTTCCGTGTTCCTA
GAPDH-R	TGTCATCATACTTGGCAGGTTT
U6-F	TGCTCGCTTCGGCAGCACAT
U6-R	CTTGCGCAGGGGCCATGCTA
MT RNR1-F	CCTCCCCAATAAAGCTAAAA
MT RNR1-R	GCTATTGTGTGTTCAGATAT
β-actin-F	AGTGTGACGTGGACATCCGCAAAG
β-actin-R	ATCCACATCTGCTGGAAGGTGGAC
HGF-F	TAGGCACTGACTCCGAACA
HGF-R	AGGAGATGCAGGAGGACAT
c-MET-F	AGTGAGAAGGCTAAAGGAAACG
c-MET-R	TTTGGACCGTCAAGAAGTAAAT
**Primers for UCA1 cloning**	
UCA1-pLVX-F	CGGAATTCTGACATTCTTCTGGACAATGAGT
UCA1-pLVX-R	CGGGATCCCTGACTCTTTTAGGAAGATTTCT
**Pimers for construction luciferase reporter vectors**	
UCA1-pmirGLO-F	GCTCTAGATGACATTCTTCTGGACAATGAGT
UCA1-pmirGLO-R	TTTTCCTTTTGCGGCCGCCTGACTCTTTTAGGAAGATTTCT
HGF-pmirGLO-F	GCTCTAGAACCTGAATGCCATGTGGGCCAT
HGF-pmirGLO-R	TTTTCCTTTTGCGGCCGCTGATCATAGAAAAGTTTATTATTTTTAAAAG
c-MET-pmirGLO-F	GCTCTAGATGCTAGTACTATGTCAAAGCAAC
c-MET-pmirGLO-R	TTTTCCTTTTGCGGCCGCTTGCATCACTTTACTTTAATTGCAT

UCA1-pmirGLO-M-F	GTacaaacaaGTTAGAGGGCTTGGGACATTTCACT
UCA1-pmirGLO-M-R	GCCCTCTAACttgtttgtACATACTTTGTGTTGTCCTGGATGC
HGF-pmirGLO-M-F	CCTcaaacaaATTAACCAAATATTTTTAAAAATATTTCCTC
HGF-pmirGLO-M-R	GGTTAATttgtttgAGGTAAGATATCTTAGTATAAAACTTGGACAG
c-MET-pmirGLO-M-F	CCTTTTAAtacaaacaaTGTTTTTTGAGACAGGATCTCACTCT
c-MET-pmirGLO-M-R	ttgtttgtaTTAAAAGGGAATGTTTACCTAATGGG
**Primers used for Sanger sequencing of KRAS, NRAS and BRAF**	
KRAS-exon2-F (Codon G12, G13)	TCAAAGAATGGTCCTGCACC
KRAS-exon2-R (Codon G12, G13)	AGGCCTGCTGAAAATGACTG
KRAS-exon3-F (Codon Q61)	ATTATTTATGGCAAATACACAAAG
KRAS-exon3-R (Codon Q61)	TCTCCCTTCTCAGGATTC
KRAS-exon4-F (Codon A146)	GAGAGAAAAACTGATATATTAAATGAC
KRAS-exon4-R (Codon A146)	TTCTAGAACAGTAGACACAAAAC
KRAS-exon4-F (Codon K117)	CTGAGCCTGTTTTGTGTCTACTG
KRAS-exon4-R (Codon K117)	TTTTATGACAAAAGTTGTGGACAGG
NRAS-exon2-F (Codon G12, G13)	CCTCTATGGTGGGATCATATTC
NRAS-exon2-R (Codon G12, G13)	CTGATTACTGGTTTCCAACAG
NRAS-exon3-F (Codon Q61)	CCTTCGCCTGTCCTCATGTA
NRAS-exon3-R (Codon Q61)	TTGAACTTCCCTCCCTCCCT
NRAS-exon4-F (Codon A146)	AATATGAATATGGATCACATCTCTA
NRAS-exon4-R (Codon A146)	ATGGTGCTAGTGGGAAACAAGTGTG
NRAS-exon4-F (Codon K117)	TAACTCTTGGCCAGTTCGTGG
NRAS-exon4-R (Codon K117)	AGGGAGCAGATTAAGCGAGTA
BRAF-exon11-F (Codon G465, G468, Y472)	AAAAGTTGTTAAACATATCCTATT
BRAF-exon11-R (Codon G465, G468, Y472)	GGGACTCGAGTGATGA
BRAF-exon15-F	GTAACTCAGCAGCATCTCAGG

BRAF-exon15-R	GCTTGCTCTGATAGGAAAATGAG

Supplementary Table 2. Clinic-pathological characteristics of metastatic CRC patients with paired specimen pre- and post-cetuximab treatment

No.	Gender	Age	Primary site	Metastatic sites	Differentiation[1]	Cetuximab regimen[2]	Best response to cetuximab[3]	Site of specimen (pre)	Site of specimen (post)
1	M	55	rectosigmoid	liver	G2	FOLFOX4+cetuximab	PR	rectum	rectum
2	F	36	left colon	liver, lung, peritonealcavity	G1-G2	FOLFOX4+cetuximab	PR	sigmoid	liver lesion
3	M	67	left colon	liver, lung	G2	FOLFOX4+cetuximab	PR	sigmoid	sigmoid
4	M	59	left colon	left ovary and adnexa, lung	G1	FOLFIRl+cetuximab	SD	sigmoid	left adnexal mass
5	M	73	rectum	liver	G2	FOLFOX4+cetuximab	PR	rectum	liver lesion
6	M	80	transverse colon	liver, bone	G3	Cetuximab	SD	transverse colon	transverse colon
7	F	52	left colon	liver	G2-G3	FOLFIRI+cetuximab	SD	left colon	liver lesion
8	F	45	left colon	liver	G1	FOLFOX4+cetuximab	PR	sigmoid	liver lesion
9	M	42	right colon	liver, lung, peritoneal cavity	G2	FOLFIRI+cetuximab	SD	hepatic flexure of colon	abdominal wall mass
10	F	58	left colon	liver, lung, peritoneal cavity	G2	mFOLFOX6+cetuximab	SD	sigmoid	omental mass

1. G1, well-differentiated, G2, moderately differentiated, G3, poorly differentiated.

2. Cetuximab 400 mg/m^2 initial dose followed by 250 mg/m^2 weekly thereafter with cetuximab dose intensity>90% were given to all subjects.

Chemotherapy regimens: FOLFOX4 or mFOLFOX6, modified FOLFOX6 (5-fluorouracil, leucovorin, and oxaliplatin).

3. PR, partial response; SD, stable disease.

Supplementary Table 3. The antibodies used in the present study

Name	Company	Code
p-MET	Santa Cruz	sc-101736
MET	Santa Cruz	sc-514148
p-p85	CST	4228
p85	CST	4292
p-AKT (Ser^{473})	CST	4060
AKT	CST	4691
p-p44/42 MAPK (Thr^{202}, Tyr^{204})	CST	4370
p44/42 MAPK	CST	4695
β-actin	Santa Cruz	sc-47778
TR Monoclonal Antibody	Proteintech	67728-1-Ig
Goat Anti-Rabbit IgG-FITC	Proteintech	SA00003-2
